# Epaology and the importance of context

**DOI:** 10.1007/s40037-020-00638-5

**Published:** 2020-12-02

**Authors:** F. Scheele

**Affiliations:** Amsterdam UMC, Amsterdam, The Netherlands

Van Enk and Ten Cate have provided an interesting perspective concerning entrustment [1]. Entrustment is part of the Entrustable Professional Activity (EPA) concept described by Ten Cate and others. Since 2007, the EPA has been introduced in many countries and several articles were published that deepen our understanding of why EPAs may be used and of the potential benefits and pitfalls in practice. In their perspective, Van Enk and Ten Cate recognize that entrustment is used both in retrospective and prospective assessments. In retrospective assessments, the assessor records how much supervision was given while the resident was performing a professional activity. In prospective assessments, often a team of assessors decides whether we can entrust the resident for the future. The authors raised the question whether professional ‘gut feeling judgement’ without clearly defined standards and measures is appropriate for assessors concerned with entrustment. Moreover, they propose further research into the language used for subjective assessments. They argue that ‘to limit evidence in assessment only to knowledge that can be fully and formally languaged would be naïve and would impoverish assessment’. In this commentary I shall take a step back and reflect on the need for uniform interpretation of the EPA concept. I shall argue that there is no universal truth and the EPA concept is best adapted to a national or even local context, as long as it optimally supports teaching and assessment. The same applies to the use of subjective assessments for entrustment.

In 2005, Olle ten Cate first proposed the concept of entrustment of professional activities in reaction to the granularity of competency frameworks and the divergence between educational theory and clinical practice [2]. In 2007, Olle and I wrote an article in which we placed the concept in a clinical context [3]. This article was written at a strategic level, and attributes of the EPA concept were based on the Obstetrics and Gynaecology program in the Netherlands. Attributes comprised a careful selection of concrete critical clinical activities, the connection of general competencies with clinical activities and a more holistic assessment focus on these clinical activities rather than on separate competencies. The clinical activities chosen would represent the specialism and cover a wide range of competencies as defined in competency frameworks. The entrustment of the chosen clinical activities would allow for stepwise independent practice during residency and a flexible duration of rotations within a training program, depending on learning curves. A nationwide and later on worldwide implementation was started and several issues emerged. For example, the issue of risk for prejudice and discrimination when professional judgement is used in assessment procedures for entrustment, or the legal aspects of independent resident performance in some jurisdictions, as mentioned by Van Enk and Ten Cate. Another issue is the question whether entrustment is meant for all clinical circumstances or only for straightforward clinical cases. The issues arising when a concept is primarily developed in a certain context and subsequently used in other contexts is well known in philosophy of science [4] and in change management literature. Users of the concept may adjust it to their own purposes by means of re-invention [5].

My colleagues and I are investigating the contextual translation of the EPA concept in the Netherlands and work is under review at this journal. The use of EPAs is mandatory in the Netherlands, and each specialism has a national curriculum which must describe which EPAs are selected and how EPAs must be used for teaching. Different specialisms give a different meaning to EPAs in their curricula, e.g. some focus only on a selection of critical clinical activities suitable for the core of a training program, while other focus more on an EPA’s suitability for entrustment. Curriculum designers pick from the EPA concept what suits them and what is valuable for medical education in their situation. This is recognized at the level of specialisms and curriculum design, but it is also recognized in the way program directors and residents use the EPA concept within their local context and personal value systems. An example of such a value system is that for the sake of patient safety, some program directors require far more extensive proof of competence before they entrust a trainee to do risky treatments than other program directors do. Is one focus and attributed meaning better than another one? I believe that the perfection of the EPA concept will prove a quest for a universal truth rather than a man-made, subjective truth based on local context. The universal truth fits in a positivist paradigm, whereas the social constructivist’s paradigm would expect the truth to be dependent on stakeholders and context. The original manuscripts were written at a strategic level. In my opinion local versions of EPA implementation will differ. The truth is in the eye of the beholder.

The entrustment concept is important and appealing. Investigations and discussions about the use of EPAs, such as the article by Van Enk and Ten Cate, are helpful to increase the knowledge of how the strategic concept may be effective in various ways in the reality of medical education. In our Dutch context, there have been various implementations of the original concept in medical education. It is important that we study the EPA implementations in different contexts so that we develop a better understanding of the relationship between the context in which the EPA is applied, the mechanisms by which EPA work and the outcomes the EPA implementation produces. When used without flexibility, the EPA concept has no magical power to solve issues of teaching, assessment and accountability. The magic is in how curriculum designers, clinical teachers and residents make optimal use of the EPA concept in their specific situation.
